# Tracheal intubation in critically ill patients: a comprehensive systematic review of randomized trials

**DOI:** 10.1186/s13054-017-1927-3

**Published:** 2018-01-20

**Authors:** Luca Cabrini, Giovanni Landoni, Martina Baiardo Redaelli, Omar Saleh, Carmine D. Votta, Evgeny Fominskiy, Alessandro Putzu, Cézar Daniel Snak de Souza, Massimo Antonelli, Rinaldo Bellomo, Paolo Pelosi, Alberto Zangrillo

**Affiliations:** 1 Department of Anaesthesia and Intensive Care, IRCCS San Raffaele Scientific Institute, Via Olgettina 60, 20132 Milan, Italy; 2 Università Vita-Salute San Raffaele, Via Olgettina 58, 20132 Milan, Italy; 3 Department of Anesthesia and Intensive Care, Siberian Biomedical Research Center of the Ministry of Health, Novosibirsk, Russia; 4 Department of Cardiovascular Anesthesia and Intensive Care, Cardiocentro Ticino, Lugano, Switzerland; 5 Department of Surgery. Discipline of Anesthesiology, Critical Care and Pain Medicine, Federal University of São Paulo, São Paulo, Brazil; 6 Department of Intensive Care Medicine and Anaesthesiology, Fondazione Policlinico Universitario A. Gemelli, Università Cattolica del Sacro Cuore, Rome, Italy; 7 Department of Intensive Care, Austin Hospital, Heidelberg, Australia; 8 School of Medicine, The University of Melbourne, Melbourne, Australia; 9 Department of Surgical Sciences and Integrated Diagnostics, San Martino Policlinico Hospital, IRCCS for Oncology, University of Genoa, Largo Rosanna Benzi 8, 16131 Genoa, Italy

**Keywords:** Tracheal intubation, Critically ill, Emergency department, Intensive care unit, Videolaryngoscopy, High flow nasal cannula

## Abstract

**Background:**

We performed a systematic review of randomized controlled studies evaluating any drug, technique or device aimed at improving the success rate or safety of tracheal intubation in the critically ill.

**Methods:**

We searched PubMed, BioMed Central, Embase and the Cochrane Central Register of Clinical Trials and references of retrieved articles. Finally, pertinent reviews were also scanned to detect further studies until May 2017. The following inclusion criteria were considered: tracheal intubation in adult critically ill patients; randomized controlled trial; study performed in Intensive Care Unit, Emergency Department or ordinary ward; and work published in the last 20 years. Exclusion criteria were pre-hospital or operating theatre settings and simulation-based studies. Two investigators selected studies for the final analysis. Extracted data included first author, publication year, characteristics of patients and clinical settings, intervention details, comparators and relevant outcomes. The risk of bias was assessed with the Cochrane Collaboration’s Risk of Bias tool.

**Results:**

We identified 22 trials on use of a pre-procedure check-list (1 study), pre-oxygenation or apneic oxygenation (6 studies), sedatives (3 studies), neuromuscular blocking agents (1 study), patient positioning (1 study), video laryngoscopy (9 studies), and post-intubation lung recruitment (1 study). Pre-oxygenation with non-invasive ventilation (NIV) and/or high-flow nasal cannula (HFNC) showed a possible beneficial role. Post-intubation recruitment improved oxygenation_,_ while ramped position increased the number of intubation attempts and thiopental had negative hemodynamic effects. No effect was found for use of a checklist, apneic oxygenation (on oxygenation and hemodynamics), videolaryngoscopy (on number and length of intubation attempts), sedatives and neuromuscular blockers (on hemodynamics). Finally, videolaryngoscopy was associated with severe adverse effects in multiple trials.

**Conclusions:**

The limited available evidence supports a beneficial role of pre-oxygenation with NIV and HFNC before intubation of critically ill patients. Recruitment maneuvers may increase post-intubation oxygenation. Ramped position increased the number of intubation attempts; thiopental had negative hemodynamic effects and videolaryngoscopy might favor adverse events.

**Electronic supplementary material:**

The online version of this article (doi:10.1186/s13054-017-1927-3) contains supplementary material, which is available to authorized users.

## Background

Critically ill patients frequently require tracheal intubation in the intensive care unit (ICU), in the emergency department (ED), or during in-hospital emergency in general wards [1–3]. Critically ill patients differ from elective surgical patients intubated in the operating theatre: often they present with severe respiratory failure, hemodynamic instability, increased sensitivity to the side effects of sedatives, recent food intake, and cardiac or cerebrovascular diseases [2]. Airway management outside the operating theatre has a high rate of major complications, such as severe hypoxia, hemodynamic collapse, cardiac arrest, and death [1, 3–6]. Moreover, the incidence of difficult intubation is high when compared to elective intubation in the operating room [1, 7, 8]. Lack of training, supervision and assistance, failure to identify patients at risk, failure to plan and carry out a backup strategy if required, and deficiency in available equipment, all are the most relevant modifiable risk factors [6]. Other factors that could increase the incidence of adverse events include the emergent requirement of tracheal intubation preventing adequate preparation, and the commonly limited physical space around ICU beds. Accordingly, interventions to improve everyday practice, in particular pre-oxygenation and first-attempt success rate, have been proposed and evaluated individually or as combined in bundles [2, 3, 9]. However, these protocols are based mostly on expert opinions, low-quality retrospective or before-after studies, or are derived from guidelines developed for elective intubation in the operating theatre. So far, there has not been a systematic review focused on randomized controlled trials (RCTs).

We performed a systematic review of RCTs evaluating any drug, technique, or device aimed at improving the success rate or the safety of tracheal intubation in critically ill patients performed in ICU, ED or general ward settings. When feasible, we also performed a meta-analytic assessment of these findings.

## Methods

### Search strategy

PubMed, BioMed Central, Embase and the Cochrane Central Register of Clinical Trials were searched for pertinent studies (updated 13 November 2017) by five investigators (LC, MB, OS, CV, and CDSS). The full PubMed/Medline search strategy is reported in Additional file 1: Figure S1. The references of retrieved articles were checked for further studies. Moreover, the investigators scanned pertinent reviews to detect further studies. No language restriction was enforced.

### Study selection

References obtained from databases and the literature were first independently examined at title/abstract level by six investigators (LC, MB, OS, CV, AP, and CDSS), with disagreement resolved by consensus under supervision of two investigators (GL and AZ) and, if potentially pertinent, full articles were retrieved.

The following inclusion criteria were employed for potentially relevant studies: (a) tracheal intubation in critically ill patients; (b) RCT; (c) study performed in adult patients in the ICU, ED or general ward; and (d) study published in the last 20 years in a peer-reviewed journal.

Exclusion criteria included pre-hospital or operating theatre settings and studies based on simulation. Two investigators (LC and GL) selected studies for the final analysis, independently assessing compliance with the selection criteria. Divergences were resolved by consensus.

### Data abstraction and study characteristics

Standardized forms were used to extract data with disagreements resolved by discussion or involving a third reviewer when required. Data, which were extracted following the patient, population or problem, intervention, comparison, outcomes (PICO) approach, included first author, publication year, characteristics of patients and clinical settings (the population), intervention details, comparators, relevant outcomes (e.g. indicators of efficacy or safety), and risk of bias. To assess the risk of bias, we used the Cochrane Collaboration’s Risk of Bias tool.

### Statistical analysis

We pooled estimates of treatment effects for each outcome by random-effects model meta-analysis using the inverse variance for binary data and Mantel-Haenszel methods for continuous data. We used the random-effects model because we anticipated that studies would include patients from varied populations, and investigators with different experience for intubation, thereby resulting in the estimation of heterogeneous intervention effects. We report continuous outcomes as mean difference and dichotomous outcomes as risk ratios (RRs) with their 95% confidence interval (CI). When continuous variables were analyzed as median and interquartile range or CI we transformed the data using the following formula: mean = median; with interquartile range standard deviation = (3rd quartile - 1st quartile)/1.35; with CI standard deviation = √sample size x (upper limit – lower limit)/3.92, to avoid losing data. We assessed heterogeneity using the *I*^2^ statistic. We also assessed the *p* value for the *I*^2^ statistic to determine the strength of evidence for heterogeneity. In accordance with Cochrane guidance, we did not analyze publication bias because our search identified fewer than ten studies for each data comparison. We compared treatment effects across subgroups using a test for interaction. We performed the analyses using an intention-to-treat approach. We conducted two-tailed statistical tests and set the probability of type I error at 0.05. All calculations and graphs were performed using Review Manager (RevMan, Version 5.3, Copenhagen: The Nordic Cochrane Centre, The Cochrane Collaboration, 2014). The protocol had been registered in the Prospero database (CRD42017068989).

## Results

Database searches and scanning of references yielded 880 articles. From these we finally identified 22 randomized clinical trials for inclusion in seven areas of interest (Additional file 1: Figure S1): use of a pre-procedure check-list (1 study) [10], pre-oxygenation and apneic oxygenation (6 studies) [11–16], sedatives (3 studies) [17–19], neuromuscular blocking agents (NMBA) (1 study) [20], patient’s position (1 study) [21], videolaryngoscopy (9 studies) [22–30], and post-intubation recruitment maneuver (RM) (1 study) [31].

One multicenter RCT evaluated the efficacy of a verbally performed 10-item pre-intubation checklist [10] compared to no checklist in 262 enrolled critically ill patients: no difference was found in any outcome (lowest peripheral oxygen saturation (SpO2), lowest systolic blood pressure, number and length of intubation attempts, life-threatening episodes, or in-hospital mortality).

The six RCTs focusing on pre-oxygenation and apneic oxygenation were heterogeneous in treatment and comparator groups [11–16]. Pre-oxygenation refers to the administration of oxygen before induction (though some oxygen delivery devices used for pre-oxygenation can then be left on after induction or even during laryngoscopy). Apneic oxygenation refers to oxygen applied to a patient who is not spontaneously breathing (i.e. during induction-to-laryngoscopy and laryngoscopy-to-intubation periods). The characteristics and findings of these six RCTs (563 patients overall) are summarized in Table 1. The two studies applying non-invasive ventilation (NIV) for pre-oxygenation compared to standard pre-oxygenation showed positive results [11, 13] In a meta-analysis of the five studies applying high-flow nasal cannula (HFNC) [12–16], stratified for the application as pre-oxygenation tool and/or as apneic oxygenation tool, limited evidence suggests that HFNC is ineffective if used for apneic oxygenation, while it might have some efficacy in improving the levels of lower oxygen saturation, but without improving the incidence of severe desaturation if used for pre-oxygenation (Additional file 1: Figures S2 and S3).

**Table 1:** Characteristics of the five studies on pre-oxygenation techniques

1st Author	Journal, year	Setting	Patients’ characteristics	Pre-oxygenation intervention	Pre-oxygenation comparator	Primary outcome	Comments
Baillard C et al. [11]	Am J Resp Crit Care Med, 2006	ICU	Severely hypoxemic patients	Pre-oxygenation with NIV	Pre-oxygenation nonrebreather bag-valve mask driven by 15 L/min oxygen. Patients were allowed to breath spontaneously with occasional assistance	Mean drop in SpO_2_ during ETI	SpO_2_ values were significantly better in the NIV group after pre-oxygenation, during intubation, and 5 min after intubation Episodes of SpO_2_ < 80% were significantly less common in the NIV group (*p* < 0.01).
Vourc’h M et al. [12]	Intensive Care Med, 2015	ICU	Severely hypoxemic patients	Pre-oxygenation and apneic oxygenation with HFNC (maintained during laryngoscopy)	HFO by facemask followed by no supplemental O_2_ during laryngoscopy	Lowest SpO_2_ throughout intubation procedure	No significant difference in any peri-procedural oxygenation parameter. Duration of mechanical ventilation was shorter in the HFNC group.
Jaber S et al. [13]	Intensive Care Med, 2016	ICU	Severely hypoxemic patients	Pre-oxygenation with NIV plus HFNC, then apneic oxygenation with HFNC (maintained during laryngoscopy)	Pre-oxygenation with NIV plus sham HFNC, then apneic oxygenation with sham HFNC (maintained during laryngoscopy)	Lowest SpO_2_ during ETI	Lowest SpO2 during intubation higher in the intervention group. In per-protocol analysis, fewer severe desaturation episodes in the intervention group.
Simon M et al. [14]	Resp Care, 2016	ICU	Severely hypoxemic patients	Pre-oxygenation with HFNC, then apneic oxygenation with HFNC (maintained during laryngoscopy)	Bag -valve mask and no supplemental O_2_ during laryngoscopy	Mean lowest SpO_2_ during ETI	No difference at any time points in SpO_2_ or pCO_2_, and in procedural-related complications.
Semler MW et al. [15]	Am J Resp Crit Care Med, 2016	ICU	Critically ill patients	Not standardized pre-oxygenation followed by apneic oxygenation with HFNC during laryngoscopy	Not standardized pre-oxygenation and no supplemental O_2_ during laryngoscopy	Lowest SpO_2_ between induction and 2 min after ETI	No significant difference in any peri-procedural oxygenation parameter. No difference in short-term and hospital mortality.
Caputo N et al. [16]	Acad Emerg Med, 2017	ED	Critically ill patients	Standard 3-min pre-oxygenation followed by apneic oxygenation with HFNC during laryngoscopy	Standard 3-min pre-oxygenation and no supplemental O2 during laryngoscopy	Average lowest SpO2 during apnea and in the following 2 minutes	No difference in lowest average SpO2, no difference in SpO2 at any time-point, no difference in the rates of moderate or severe desaturation episodes.

*Abbreviations*: *ICU* intensive care unit, *ETI* endotracheal intubation, *NIV* non invasive ventilation, *HFNC* high-flow nasal cannula, *HFO* high-flow oxygen, *SpO*_*2*_ peripheral oxygen saturation, *PaO*_*2*_ arterial oxygen pressure, *ED* emergency department

Three studies compared sedatives. Sivilotti et al. compared thiopental, fentanyl and midazolam (together with NMBA) during rapid sequence induction in the ED in 86 critically ill patients [17]: thiopental slightly shortened the time to intubation but was associated with lower reduction in systolic blood pressure compared to fentanyl and midazolam, while midazolam was associated with an increase in heart rate compared to fentanyl and thiopental. In the second study, alfentanil, sufentanil, and fentanyl were compared during rapid sequence intubation (RSI) in the ED in 90 trauma patients [18]: no significant difference in hemodynamic parameters was observed. Finally, Jabre et al. evaluated etomidate versus ketamine in 469 critically ill patients in 12 EDs and 65 ICUs in France [19]: no difference in intubation conditions or intubation-related adverse events was found.

In the only RCT on NMBA, succinylcholine was compared to rocuronium for RSI in 401 ICU patients [20]: no difference was observed in intubation conditions, rate of success of first attempt, and oxygen desaturation episodes, but the duration of the intubation sequence was on average 14 seconds shorter with succinylcholine.

One multicenter trial compared the sniffing position (entire bed flat, with the patient’s head elevated) and ramped position (upper half of the bed raised at an angle of 25° and the neck extended to have the patient’s face parallel to the ceiling) during laryngoscopy in 260 patients [21]. The sniffing position allowed a better view of the glottis across the full range of body mass indices and prior level of experience, and reduced the number of intubation attempts; however, it did not improve oxygenation, hemodynamic, or other clinical outcomes.

**Fig. 1:**
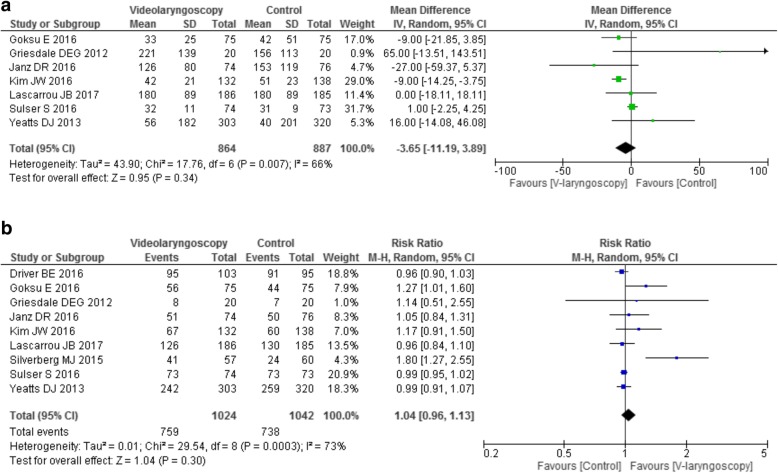
Videolaryngoscopy vs. direct laryngoscopy: forest plot for intubation time (**a**) and for first-attempt successful intubation (**b**)

Nine studies compared different models of videolaryngoscopy to direct, traditional laryngoscopy in different conditions and settings in 2069 patients (Table 2) [22–30]. Time to intubation and first-attempt success rate were the most relevant reported outcomes. Videolaryngoscopy did not shorten the time to intubation (Fig. 1a), nor the first-pass success rate (Fig. 1b), even when evaluating the studies according to the greater or lesser experience of the operators, or according to the setting (ICU versus ED), or the model used (hyper-angulated vs non-hyper-angulated) (Additional file 1: Figures S4 − S6). Four studies [22, 25, 27, 29] analyzed the subgroups with anticipated difficult airways: no study found a difference in the outcomes. The two largest trials found an increased incidence of severe complications in post-hoc analyses when videolaryngoscopy was employed: Yeatts et al. reported longer duration of the intubation procedure, greater incidence of severe desaturation episodes and highest mortality rate in the group with severe head injury, while Lascarrou et al. reported an increased incidence of life-threatening complications [22, 29].

**Table 2:** Characteristics of the nine studies comparing videolaryngoscopy to direct laryngoscopy

1st author	Journal, year	Setting	Patients’ characteristics	Personnel performing ETI	Videolaryngoscope model	Primary outcome	Comments
Yeatts DJ et al. [22]	J of Trauma and Acute Care Surg, 2013	Trauma resuscitation unit	Adult critically ill trauma patients	Emergency medicine residents, anesthesiology residents, attending anesthesiologists, nurse anesthetist	GlideScope	Survival to hospital discharge	No difference in the subgroup with anticipated difficult airways. Higher incidence of severe desaturation and worse mortality in the subgroup of head-injured patients intubated with videolaryngoscope
Griesdale DEG et al. [23]	Can J Anesth, 2012	ICU, ordinary ward, ED	Adult critically ill patients	Medical students or non-anesthesiology residents	GlideScope	Number of intubation attempts	No difference in intubation attempts. Significantly better visualization in the videolaryngoscope group, but lowest SaO2 during first attempt
Kim JW et al. [24]	Resuscitation, 2016	ED	Adult patients in cardiac arrest	Experienced intubators	GlideScope	Success rate of ETI by the intubator	No difference in the incidence of esophageal intubation and tooth injury. Chest compression interruption during CPR were longer in the direct laryngoscopy group
Goksu E et al. [25]	Turk J Emerg Med, 2016	ED	Blunt trauma patients	Residents and attending physicians of the ED	C-MAC	Overall successful intubation	Better glottis visualization and decreased esophageal intubation rate with videolaringoscopy. No difference in success rate even separating easy and difficult intubations
Janz DR et al. [26]	Crit Care Med, 2016	ICU	Adult critically ill patients	Pulmonary and critical care fellows	McGrath Mac or GlideScope or Olympus	Intubation on first attempt, adjusted for the operator’s previous experience	Better glottis visualization with videolaryngoscopy. No other differences
Driver BE et al. [27]	Acad EmergMed, 2016	ED	Adult critically ill patients	Senior residents	C-MAC	First-pass success rate	No difference in duration of first attempt, aspiration, hospital length of stay. No difference in success rate in the subgroup with anticipated difficult airways
Sulser S et al. [28]	Eur J Anaesth, 2016	ED	Adult critically ill patients	Experienced anesthesia consultants	C-MAC	First attempt success rate	Better glottis visualization in the videolaryngoscopy group. No difference in desaturation episodes or complications
Lascarrou JB et al [29]	JAMA, 2017	ICU	Adult critically ill	ICU physicians	McGrath Mac	Successful first-pass intubation	Better glottis visualization, but higher number of life-threatening complications with videolariyngoscopy. No difference in success rate even stratified for operator experience and expected difficult airways. No difference in number of intubation
Silverberg MJ et al. [30]	Crit Care Med, 2015	ICU and ordinary wards	Adult critically ill patients	Pulmonary and critical care fellows	GlideScope	First-attempt success rate	Better glottis visualization and lower number of attempts in the videolaryngoscopy group. No difference in overall complications rate. Neuromuscular blocking agents were not used

*Abbreviations*: *ICU* intensive care unit, *ETI* endotracheal intubation, *SpO*_*2*_ peripheral oxygen saturation, *CP*R cardiopulmonary resuscitation

Finally, Constantin JM et al. evaluated the efficacy of an RM (40 cmH_2_O for 30 seconds) immediately after successful RSI in 40 ICU hypoxemic patients [31]. The RM group had significantly higher arterial pO_2_ both 5 and 30 minutes after the RM; no difference in hemodynamic parameters was observed, although one RM was interrupted due to hypotension.

## Discussion

### Key findings

We performed a systematic review focusing on RCTs evaluating drugs, techniques or devices aimed at improving the success rate or the safety of tracheal intubation in adult critically ill patients in the ICU, ED or general wards. We identified 22 trials focusing on seven different areas. Our main findings were a possible beneficial role of pre-oxygenation with NIV and/or HFNC, the negative effect of thiopental on blood pressure, and the possible efficacy of post-intubation recruitment in increasing arterial PaO_2_. HFNC for apneic oxygenation seems ineffective; the sniffing position reduced the number of intubation attempts, without improving clinically relevant outcomes. No other significant beneficial or negative effect was observed among the other evaluated interventions such as use of a checklist, choice of opioids, choice of etomidate versus ketamine, choice of rocuronium versus succinylcholine, and use of a videolaryngoscope (which on the contrary was associated with increased adverse events in four trials).

### Relationship to previous studies

Tracheal intubation of critically ill patients is a common procedure and is frequently complicated by severe adverse events, with an incidence ranging from 4.2 to 39% [1, 4–7]. To improve safety or efficacy of the procedure, standardization of the approach was proposed in the form of bundle or checklist [2, 9] including identification of patients at high risk; pre-oxygenation; monitoring, specific equipment, drugs, and algorithms. Recently, a 10-point bundle was evaluated in three ICUs: a significant decrease in severe and non-severe complications was observed [3]. However, most of the available recommendations on the topic are based on expert opinion or non-randomized studies. Furthermore, they are derived from guidelines developed for a different setting (elective intubation in the operating theatre) where airway-management-related deaths are 30-fold less common than in the ICU and ED and brain damage 60-fold less common [32]. Hence, we decided to perform a systematic review to identify the best available evidence-base on the topic, help improve daily practice and inform future research.

The only RCT comparing a pre-intubation checklist to no checklist did not find any positive effect [10]: these results are in contrast with the above mentioned before-after study [3], and in line with another observational study [9]. A different choice of items could at least in part explain the difference: the positive results were obtained in a center with extensive experience in intubation specifically in critically ill patients, while the checklist of the RCT derived from the opinions of experts in airway management and from guideline-recommended steps for intubation, not focused on critically ill patients [9]. The present systematic review could contribute to identify the most relevant items to be included in future checklists.

Pre-oxygenation of patients to be intubated has a strong rationale, extending the duration of safe apnea during laryngoscopy before the patient reaches critical levels of hypoxemia [33]. Apneic oxygenation can be complementary to pre-oxygenation techniques [34]. Pre-oxygenation before intubation of critically ill patients and, above all, of hypoxemic patients, is crucial. However, the techniques commonly applied in the operating room (spontaneous breathing of high concentration of oxygen applied by face mask for some minutes, followed by manual ventilation by a bag-valve mask) might be not effective or feasible in the deranged physiology of ICU and ED patients. Our findings suggest that HFNC might improve pre-oxygenation; on the other hand, apneic oxygenation with HFNC seems ineffective. These findings are in line with a recent meta-analysis [35] also including a non-randomized trial: apneic oxygenation with HFNC reduced severe desaturation in elective intubation in the operating room (OR), but not in ICU patients with respiratory failure. On the contrary, NIV applied for 3 minutes before laryngoscopy resulted in a better safety profile reducing the incidence of severe desaturation episodes, without NIV-related complications [11]: NIV may be regarded as a useful approach to pre-oxygenation in critically ill patients, above all in hypoxemic patients, even if it was evaluated only in a single, small study.

Three unrelated heterogeneous RCTs evaluated sedatives. The only large one evaluated etomidate versus ketamine, finding no difference [19]: a Cochrane meta-analysis also investigating non-intubation-related adverse effects of etomidate in critically ill patients concluded that its use is not associated with worsening of mortality, organ dysfunction or resource utilization, even if it negatively affects adrenal gland function [36]. Unfortunately, and surprisingly, no RCT evaluated the very commonly used sedative propofol in this context.

Marsch at al. found that succinylcholine and rocuronium for rapid sequence intubation (RSI) are equivalent, even if the duration of intubation was longer with rocuronium [20]. It should be noted that the study did not consider the potential beneficial role of the antagonist sugammadex when using rocuronium [37]. Moreover, even if RSI is commonly considered the technique of choice in critically ill patients [38], graded sedation intubation not using NMBA has also been proposed and applied [28, 37–40]. Unfortunately, the two approaches have never been compared.

In a multicenter study, the sniffing position during laryngoscopy improved the rate of first-attempt success rate compared to the ramped position, without improving oxygenation and hemodynamic parameters [21]. These findings are in contrast with previous studies, in which the ramped position provided a better view of the glottis [41–43] and seemed to improve pre-oxygenation [44, 45]. However, all previous studies were performed in the operating room in elective patients.

Tracheal intubation of critically ill patients is associated with increased frequency of difficult intubation compared with elective intubation in the operating theatre [1, 7, 8]. Furthermore, multiple attempts at intubation are associated with a higher risk of severe complications, due to the limited physiological reserve of these patients [5]. Our findings suggest that videolaryngoscopes do not perform better than traditional direct laryngoscopy across a wide range of conditions, even if they could offer better visualization of the glottis. On the contrary, four trials found an increased incidence of severe complications when videolaryngoscopy was used [22, 29]. Our results are in line with two recent meta-analyses of randomized trials on videolaryngoscopy limited to the ICU setting [46, 47], and in contrast with a previous meta-analysis on videolaryngoscopy in the ICU setting also including non-randomized trials and reporting an increased first-pass success rate (but not a reduction in complications) [48]. Moreover, a recent meta-analysis including 64 studies (61 performed in elective surgical patients) concluded that videolaryngoscopy may reduce the number of failed intubations, particularly among patients with a difficult airway, but no evidence indicates that they reduce the number of intubation attempts, the incidence of hypoxia or respiratory complications, and/or the time required for intubation [49]. Available evidence does not support the routine use of videolaryngoscopy in critically ill patients; moreover, videolaryngoscopy might be associated with an increased incidence of severe adverse events.

In a small RCT, an RM after intubation improved oxygenation at 5 and 30 minutes, without any other difference in hemodynamic parameters. RM has been evaluated mainly in acute lung injury and acute respiratory distress syndrome, but its role is still debated [50]. Based on this single RCT, RM could be useful after intubation in hypoxemic patients, even if its effect declines after 30 minutes.

### Implications of study findings

Our findings imply that, in hypoxemic patients, time permitting, pre-oxygenation by NIV and/or HFNC could offer the best safety profile; post-intubation RM can further enhance arterial oxygenation. The sniffing position might be the position of choice for laryngoscopy. Thiopental should be avoided, above all in hemodynamically unstable patients.

### Strengths and limitations

The present study has several strengths. It is the first systematic review comprehensively evaluating all steps of tracheal intubation in critically ill patients in every setting (the ICU, ED, and general wards). Moreover, it is based only on evidence from RCTs. Our findings are relevant to the development of evidence-based algorithms on the topic. Furthermore, we identified the lack of data in many areas, hopefully informing future research.

The main limitation of the present systematic review is its inability to offer robust suggestions about crucial areas. In particular, no RCT evaluated the role and compared the performance of different scores to predict difficult intubation [2, 9], the best monitoring and equipment, the role of supervision, the best associated drugs (in particular the role of propofol, a commonly used sedative), the best way to face predicted and unpredicted difficult airways scenarios, the role of fiber optic bronchoscopy and supraglottic devices, the best strategy to confirm tracheal intubation, and how to prevent or treat hemodynamic instability [2, 9]. RCTs and meta-analyses cannot be the only elements guiding daily practice, as many aspects remain (and will likely remain) unexplored by RCTs. In these areas, we still depend largely on expert opinions, low-quality studies and algorithms developed for the OR. As the Fourth National Audit Project conducted in the UK on major complications of airway management concluded, airway management in the ICU and ED is still under-explored [6]. Nevertheless, our findings allow the definition of more robust evidence-based strategies and will inform future research.

## Conclusions

We identified and meta-analyzed 22 RCTs in seven different areas, evaluating drugs, techniques or devices aimed at improving the success rate or the safety of tracheal intubation in critically ill patients. The main findings were a possible beneficial role of pre-oxygenation with NIV and/or HFNC, the effect of the ramped position in increasing the number of intubation attempts, the negative impact of thiopental on blood pressure, and the possible efficacy of post-intubation RM in increasing arterial PaO_2_. No other significant effect was found in the use of a checklist, choice of drugs, neuromuscular blockers, and use of videolaryngoscopy (the latter being associated with severe adverse effects in four trials). Further research in this poorly explored area is required.

## Additional file

Additional file 1: Search strategy, flow chart of the systematic review, supplemental figures (forest plots). (DOCX 146 kb)
