# Correction to: Tracheal intubation in critically ill patients: a comprehensive systematic review of randomized trials

**DOI:** 10.1186/s13054-019-2634-z

**Published:** 2019-10-21

**Authors:** Luca Cabrini, Giovanni Landoni, Martina Baiardo Redaelli, Omar Saleh, Carmine D. Votta, Evgeny Fominskiy, Alessandro Putzu, Cézar Daniel Snak de Souza, Massimo Antonelli, Rinaldo Bellomo, Paolo Pelosi, Alberto Zangrillo

**Affiliations:** 1 Department of Anaesthesia and Intensive Care, IRCCS San Raffaele Scientific Institute, Via Olgettina 60, 20132 Milan, Italy; 2 Università Vita-Salute San Raffaele, Via Olgettina 58, 20132 Milan, Italy; 3 Department of Anesthesia and Intensive Care, Siberian Biomedical Research Center of the Ministry of Health, Novosibirsk, Russia; 4 Department of Cardiovascular Anesthesia and Intensive Care, Cardiocentro Ticino, Lugano, Switzerland; 5 Department of Surgery, Discipline of Anesthesiology, Critical Care and Pain Medicine, Federal University of São Paulo, São Paulo, Brazil; 6 Department of Intensive Care Medicine and Anaesthesiology, Fondazione Policlinico Universitario A. Gemelli, Università Cattolica del Sacro Cuore, Rome, Italy; 7 Department of Intensive Care, Austin Hospital, Heidelberg, Australia; 8 School of Medicine, The University of Melbourne, Melbourne, Australia; 9 Department of Surgical Sciences and Integrated Diagnostics, San Martino Policlinico Hospital, IRCCS for Oncology, University of Genoa, Largo Rosanna Benzi 8, 16131 Genoa, Italy

**Correction to: Crit Care (2018) 22:6**

**https://doi.org/10.1186/s13054-017-1927-3**

In the publication of this article [1], there was an error in a contributors Family Name. This has now been updated in the original article.

The error: Baiardo Radaelli

Should instead read: Baiardo Redaelli
