# Supplementary material for: Tracheal intubation in critically ill patients: a comprehensive systematic review of randomized trials
Source: Crit Care. 2018 Jan 20;22:6. doi: 10.1186/s13054-017-1927-3 (PMC5775615; doi:10.1186/s13054-017-1927-3)

# Additional file 1

Search strategy:

(intubation[tiab]) AND (laryngoscop*[tiab] OR bronchoscop*[tiab] OR "supraglottic airway device"[tiab] OR “fiber optic”[tiab] OR macintosh[tiab] OR “tracheal tube”[tiab] OR “endotracheal tube”[tiab] OR “positive-pressure respiration”[tiab] OR “continuous positive airway pressure”[tiab] OR “neuromuscular blocking agents”[tiab] OR “neuromuscular blockade”[tiab] OR sedat*[tiab] OR “oxygen inhalation therapy”[tiab] OR insufflation[tiab] OR “high flow nasal cannula”[tiab] OR “pre-oxygenation”[tiab] OR “noninvasive ventilation”[tiab]) AND (acute [tiab] OR "critical care"[tiab] OR "critical illness"[tiab] OR "critically ill"[tiab] OR "intensive care"[tiab] OR ICU[tiab] OR emergen*[tiab] OR “out of hospital”[tiab] OR ALI[tiab] OR “lung injury”[tiab] OR “respiratory distress syndrome"[tiab] OR ARDS[tiab] OR “respiratory insufficiency”[tiab] OR “respiratory failure”[tiab] OR “airway obstruction”[tiab] OR asphyxia[tiab] OR “difficult airway”[tiab] OR hypoxia[tiab] OR hypoxemia[tiab] OR “cardiac arrest"[tiab] OR asystole[tiab] OR ECMO[tiab] OR “extracorporeal membrane”[tiab] OR “extracorporeal life support”[tiab] OR “extracorporeal circulation”[tiab] OR anaphylaxis[tiab] OR sepsis[tiab] OR infection[tiab] OR pneumonia[tiab]) AND ("randomized controlled trial"[tiab] OR "controlled clinical trial"[tiab] OR "randomized controlled trials"[tiab] OR blind*[tiab] OR "clinical trial"[tiab] OR "clinical trials"[tiab] OR placebo*[tiab] OR random*[tiab]) NOT (animal[mh] NOT human[mh]) NOT (comment[pt] OR editorial[pt] OR meta-analysis[pt] OR practice-guideline[pt] OR review[pt] OR pediatrics[mh] OR paediatric[tiab] OR infants[tiab] OR children[tiab])**Figure S1** Flow chart of the systematic review

Further exclusions

- 32 did not meet inclusion/exclusion criteria

- 27 simulation studies

-16 elective procedures or non pertinent setting

- 7 non randomized controlled trials

- 1 unable to retrieve the full-text

-

Full text examination

303 papers excluded due to non pertinent setting (out of hospital, non emergency department, non intensive care unit)

198 papers excluded because they were simulation studies

172 papers excluded due to non critically ill population

82 papers excluded because not dealing with endotracheal intubation

20 papers excluded due to non randomized controlled trial design

22 RCTs:

-1 use of a check-list

9 videolaringoscopy

-6 preoxygenation

- 3 sedatives

-1 neuromuscolar blocking agents

-1 patient’s positioning

-1 post intubation recruitment

Articles title/abstract examination

880 papers initially identified by the search strategy

**Figure S2:** High flow nasal cannula vs control: forest plot for lowest mean SpO2 during intubation

**
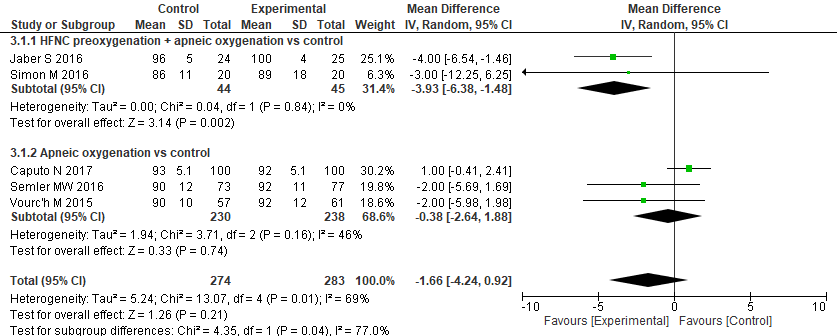
**

**Figure S3:** High flow nasal cannula vs control: forest plot for severe desaturation (SpO2 < 80%) rate

**
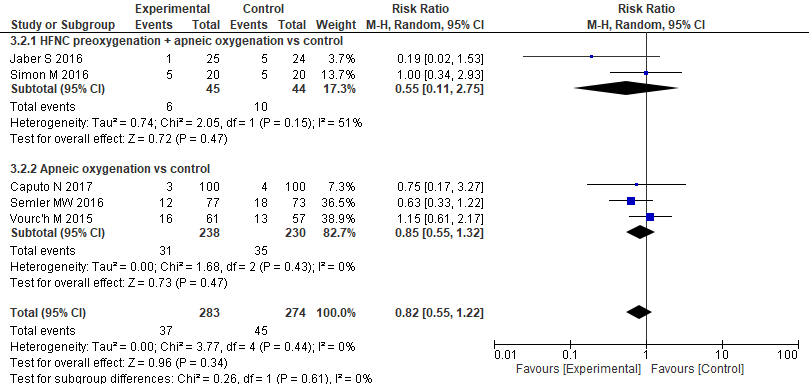
**

**Figure S4** Forest plot for first-attempt successful intubation (videolaryngoscopy vs direct laryngoscopy): comparison according to level of experience in tracheal intubation.

**Figure S5** Forest plot for first-attempt successful intubation (videolaryngoscopy vs. direct laryngoscopy): comparison according to the setting, Intensive Care Unit vs. Emergency Department.
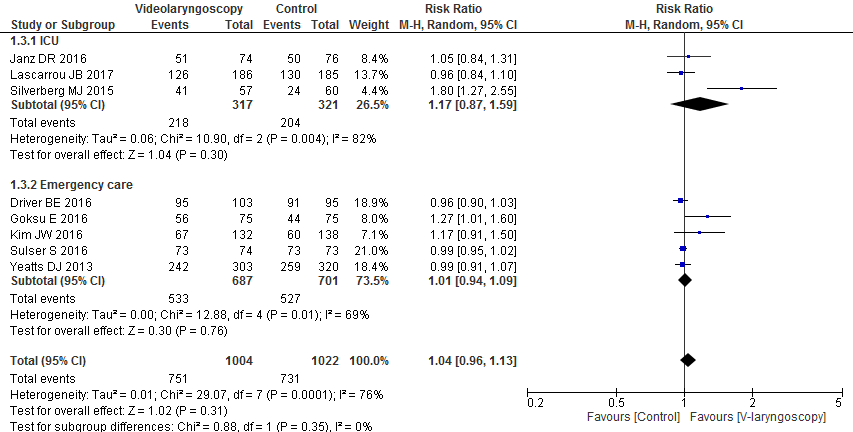


**Figure S6** Forest plot for first-attempt successful intubation (videolaryngoscopy vs. direct laryngoscopy): comparison according to the model of videolaryngoscope, Glidescope vs. C-MAC.


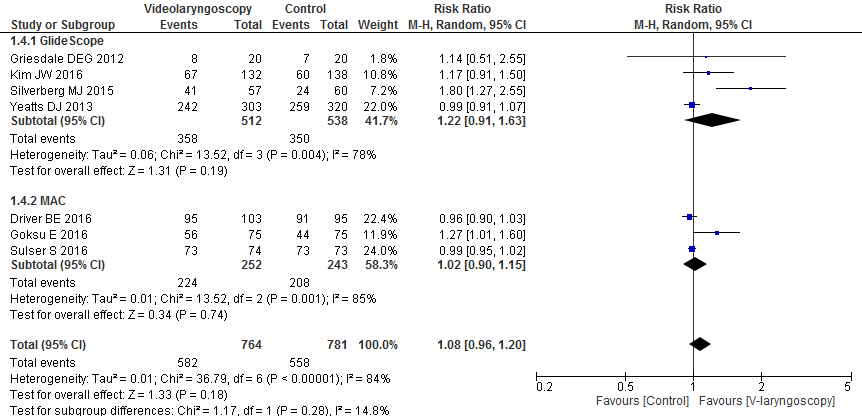

Supplement: Supplementary file 1 — Search strategy, flow chart of the systematic review, supplemental figures (forest plots). (DOCX 146 kb) [file 13054_2017_1927_MOESM1_ESM.docx]
